# Window for Dioxin Damage: Sperm Quality in Men Born after the Seveso Disaster

**DOI:** 10.1289/ehp.119-a219b

**Published:** 2011-05

**Authors:** Julia R. Barrett

**Affiliations:** **Julia R. Barrett**, MS, ELS, a Madison, WI–based science writer and editor, has written for *EHP* since 1996. She is a member of the National Association of Science Writers and the Board of Editors in the Life Sciences

Animal studies demonstrate that endocrine-disrupting chemicals such as dioxin and dioxin-like compounds are particularly damaging when exposure occurs during prenatal and early-life development. A new study links dioxin exposure within this time frame in humans to reduced sperm quality in adult males **[*EHP*** **119(5):713–716, Mocarelli et al.]**.

In July 1976, a trichlorophenol plant explosion near Seveso, Italy, resulted in dioxin contamination of the surrounding area. Thirty-nine men who were born near Seveso between March 1977 and January 1984 made up the exposed group in the current study, while 58 age-matched controls were born outside the contaminated area. Archived blood serum samples collected from the exposed men’s mothers in 1976–1977 were used to estimate the men’s prenatal dioxin exposure. All the men completed a health and lifestyle questionnaire and provided blood and semen samples. Blood samples were used for health screening tests, dioxin measurements, and hormone assays, and semen samples were analyzed for sperm motility, concentration, and morphology.

Exposed mothers had an estimated median serum dioxin concentration of 26.0 ppt at conception, whereas the median for the comparison group was estimated at 10.0 ppt. Twenty-one of the 39 exposed men were breastfed, which increased their median estimated total dioxin exposure to 40 ppt at 4–5 months of age, a critical time point for proliferation of Sertoli cells, which determine spermatogenic potential in adulthood.

Breastfed exposed men had significantly decreased sperm concentration, total sperm count, and total number of motile sperm in contrast to the 58 men in the comparison group. Compared with both the 36 breastfed comparison men and the 18 formula-fed exposed men, the 21 breastfed exposed men also had increased follicle-stimulating hormone and decreased inhibin B, a hormone pattern previously shown to be a marker for impaired spermatogenesis.

This is the first human study to show that dioxin exposure during development may permanently impair sperm production in adulthood. Current serum dioxin concentrations in the U.S. and European general populations of infants are far below levels that would trigger adverse effects. However, the study results may explain widespread low sperm counts in young men exposed during breastfeeding during times when background dioxin concentrations were 10–20 times higher than today. They also raise concerns for areas that are currently undergoing rapid industrial development and potential related contamination with dioxin and other endocrine-disrupting chemicals.

## Figures and Tables

**Figure:**
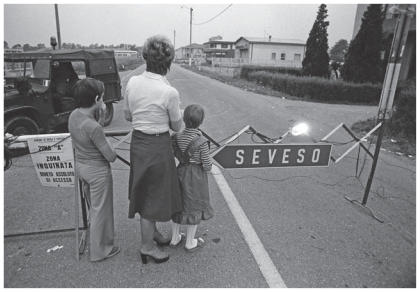
The 10 July 1976 Seveso disaster resulted in the highest dioxin exposure documented in humans and the largest cohort of dioxin-exposed women.

